# Analytical approximation for macroscopic fundamental diagram of urban corridor with mixed human and connected and autonomous traffic

**DOI:** 10.1049/itr2.12020

**Published:** 2020-12-31

**Authors:** Guojing Hu, Weike Lu, Robert W. Whalin, Feng Wang, Tor A. Kwembe

**Affiliations:** ^1^ Department of Mathematics and Statistical Sciences Jackson State University Jackson Mississippi USA; ^2^ Alabama Transportation Institute The University of Alabama Tuscaloosa Alabama USA; ^3^ Department of Civil and Environmental Engineering Jackson State University Jackson Mississippi USA; ^4^ Ingram School of Engineering Texas State University San Marcos Texas USA

**Keywords:** Mobile robots, Traffic engineering computing

## Abstract

Advances in connected and autonomous vehicles have the promise to reshape the future of the transportation system. How and when the benefits associated with automation and connectivity technology will start to impact the performance of an urban corridor is an issue of interest for traffic operators. This paper proposes an analytical capacity model for urban corridors with mixed traffic based on the concept of macroscopic fundamental diagram. The model incorporates the full spectrum of connected and autonomous vehicle penetration rates as well as the reaction times of different vehicle following patterns. The connected and autonomous vehicle platoon intensity, formulated as an exponential function of the connected and autonomous vehicle penetration rate, is also considered in the proposed analytical capacity model. Numerical experiments are conducted to verify that different reaction time settings yield disparate results. Some reaction time settings were found to cause the corridor capacity to increase monotonically with the connected and autonomous vehicle penetration rate while others led to decreases in corridor capacity with connected and autonomous vehicle penetration rates. Finally, the validity of the proposed methodology is verified via simulation tests in VISSIM 2020.

## 1 INTRODUCTION

Nowadays, connected and automated vehicle technologies are among the most emerging technologies. The Society of Automotive Engineers (SAE) defines six levels of driving automation—from level 0 of no autonomation to level 5 of full automation [1]. In addition, vehicle‐to‐vehicle (V2V) communication technology enables vehicles within the communication range to wirelessly exchange transportation information, such as speed, position, and heading, in real time. In this study, vehicles with neither automation nor V2V technology refer to human‐driven vehicles (HVs), while vehicles with automation level 5 and V2V technology refer to connected and autonomous vehicles (CAVs).

With the introduction of CAVs, the roadway system is ushering the stage of mixed traffic in which CAVs will coexist with HVs. The mixed stage has gained increasing interest among researchers, vehicle manufacturers, and policymakers because of the potential impact on many aspects of highway transportation. In particular, the increasing penetration of CAVs is expected to improve roadway capacity, but to what extent it can be increased remains an open question. According to the latest literature review [2], most studies concluded that CAVs could improve the capacities of links and intersections in the traffic system, especially when CAVs are at high levels of automation and penetration. In contrast, a few of the research opinions on the topic were skeptical and questioned whether the low penetration of CAVs may result in a potential decrease in capacity and a decline in network performance [3]. Currently, the analyses of traffic capacity in mixed traffic environment are mainly conducted on micro‐simulation platforms [4, 5, 6, 7, 8, 9, 10], while only a few are devoted to analytical approaches [11, 12, 13, 14]. These few analytical approaches mainly focus on freeways or highways, and do not involve urban street corridors with intersection signals. Therefore, a void exists in the analytical methods on studying the capacity of urban street corridors that have mixed traffic of HVs and CAVs.

To fill this void, the paper aims to provide a theoretical framework that may shed a light on how urban street capacity will evolve with the introduction of CAVs. First, we develop a triangular fundamental diagram (FD) of mixed flow that considers reaction times of different vehicle following patterns as well as CAV penetration rate and CAV platoon size. Second, given the street topology and signal timing scheme, the maximum flow and backward wave speed derived from the above triangular FD can be used to develop the macroscopic FD (MFD) of an urban street. Finally, macroscopic capacities of the urban street under different CAV penetrations are obtained from the MFDs.

The paper is organized as follows: Section 2 discusses literature that is relevant to capacity analyses of mixed traffic flows and analytical approximations for MFDs. Section 3 presents the analytical framework to derive urban street capacities under different CAV penetration rates. The numerical analysis and simulation results are presented in Section 4. Finally, Section 5 concludes the paper with a summary of findings and a brief discussion of future research directions.

## 2 LITERATURE REVIEW

### 2.1 Capacity effect of CAVs in a mixed traffic

A one hundred percent deployment of CAVs on roadways will significantly increase the capacity of the existing transportation systems due to smaller time headways between consecutive CAVs. However, there will be a long transition time from HVs to totally CAVs. According to estimates, most new vehicles will have some form of connectivity by the year 2025 [15], and 40% of vehicles traveling could be autonomous by the year 2045 [16]. During the transition period, the effect of mixed traffic containing both HVs and CAVs on the systemwide performance of transportation network is of high research interest.

The research that currently exists on this issue provides relevant insights through the simulation platform, where microscopic features of CAVs such as acceleration and headway time are incorporated [3, 5, 6]. Based on the new driving features of CAVs, innovative controlling algorithms, including CAV platooning and cooperative lane‐changing strategies, are developed and verified in the simulation platform [9, 10, 17, 18, 19]. The majority of the simulation results have verified that with the growth of CAV penetration rate, the increase of CAV platoon intensity, and the reduction of headway time, the road capacity will be improved. However, the magnitude of capacity improvement is uncertain and varies significantly with CAV technologies. For example, for CAV penetration rate from 0% to 100% on a link, capacity was increased by 2.5% in Shi et al. [8], increased by 37% in Stanek [4], increased by 90% in Liu et al. [7], increased by more than 100% in Talebpour and Mahmassani [5], and increased by 217% in Olia et al. [15, 18]. Ye and Yamamoto [6] found that different time gaps of CAVs resulted in different capacities: 1.1 seconds of CAV time gap led to 17% growth in link capacity while 0.5 seconds of CAV time gap could lead to 105% growth in link capacity.

In comparison to the extensive efforts and interest in simulation, only a few studies have attempted to use analytical models to characterize the capacity of mixed traffic, as listed in Table 1. Levin and Boyles [13] found that link capacity increases as the AV proportion increases as well as when reaction time decreases. For CAV penetration rate from 0% to 100%, Qin and Wang [11, 14] deduced about 40% increase in link capacity when the CAV reaction time is 1.1 s, and 125% increase in link capacity when the CAV penetration rate is 0.6 s. Besides CAV penetration rate and headway time, Ghiasi et al. [11] also considered CAV platoon intensity in link capacity analysis and revealed that higher CAV penetration rates and platooning intensities may not always contribute to positive changes in capacity. Similarly, Chen et al. [12] observed that CAV platoon intensity may have positive or negative impacts on lane capacity, depending on the spacing between different vehicle types. However, all the existing analytical research studies on the capacity of mixed traffic flow are concentrated on links without traffic signals. And for the urban corridors, their capacity has not been studied theoretically. Aiming to fill this research void, the paper adopts the MFD concept to evaluate the capacity of signalized urban corridors, and establishes an analytical corridor capacity framework under mixed environment.

**TABLE 1:** Analytical studies on CAV impact on road capacity in mixed traffic

Tool	Author	Scope	Consideration	Capacity effect
Analytical method	Levin, and Boyles [13]	link	AV penetration; reaction time	**Positive**: Capacity increases with the growth of AV penetration rate and the decrement of AV reaction time.
	Qin, and Wang [14]	link	CAV penetration; reaction time	**Positive**: Capacity increases with the growth of CAV penetration rate.
	Ghiasi, et al. [11]	link	CAV penetration; headway; platoon intensity	**Positive or negative**: Capacity is not always an increasing function of CAV platoon intensity, depending on the headways of different vehicle types.
	Chen, et al. [12]	link	AV penetration; spacing; platoon intensity	**Positive or negative**: Lane capacity generally increases with platoon size, but the opposite may hold: lane capacity decreases with platoon size, depending on the spacing between different vehicle types.
	This model	signalized corridor	CAV penetration; reaction time; platoon intensity	

### 2.2 Mixed corridor capacity evaluation based on MFD

At the link level, FD describes the relationship between flow, density, and speed [20]. At the network level, Godfrey [21] was the first that proposed a unimodal relationship between the average network flow and density. Such a relationship was reintroduced and verified as an MFD by Geroliminis and Daganzo [22]. Utilizing empirical data from Yokohama, Japan, Geroliminis and Daganzo [22] revealed that MFD is a reproducible and well‐defined relationship between space‐mean flow, average density, and average speed on an urban network. Since then, MFD has gained widespread popularity and considerable attention due to its outstanding ability in network controlling [23, 24, 25, 26, 27, 28, 29], traffic assignment [30, 31, 32, 33, 34], and evaluating network performance [35, 36, 37]. This paper will also adopt MFD to evaluate the corridor capacity. To analytically estimate the corridor MFD, Daganzo and Geroliminis [38] introduced the method of cuts, which was then evolved from different perspectives. For example, Leclercq and Geroliminis [39] improved the method of cuts by providing all the necessary cuts to accommodate irregular topology and signal timings, and Laval and Castrillón [40] extended the method of cuts to produce stochastic corridor MFDs. Method of cuts is featured in relating the link FD to corridor MFD, which provides a reliable basis for this paper to estimate the corridor MFD under the mixed CAV and HV environment.

## 3 METHODOLOGY

**FIGURE 1:**
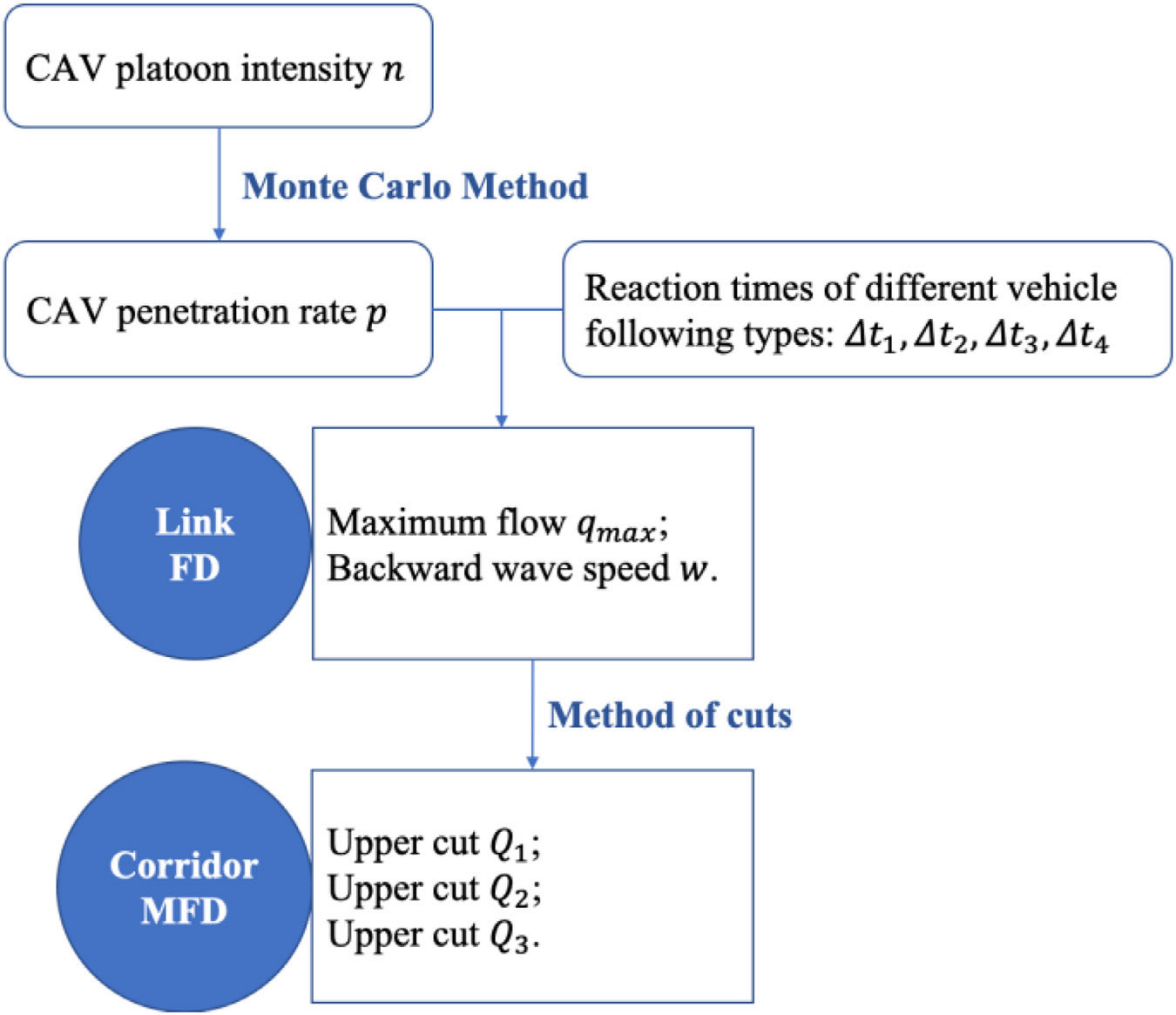
Framework of this methodology

In this section, we will first introduce the FD of a mixed traffic flow, discussing the elements (CAV penetration rate, CAV platoon intensity, vehicle reaction times) that influence the mixed traffic flow FD. We then investigate the relationship between CAV platoon intensity and CAV penetration rate through Monte Carlo Method, so as to update the mixed traffic flow FD. Afterwards, through the updated FD, an urban corridor MFD can be derived using the method of cuts. The framework of this methodology is shown in Figure 1.

### 3.1 FD of mixed traffic flow

**FIGURE 2:**
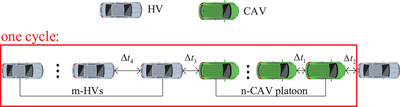
One traffic stream consisting of m‐HVs and one n‐CAV platoon

For the sake of brevity, a mixed traffic flow in a link is divided into periodically distributed traffic streams consisting of *n* CAVs as one platoon followed by *m* HVs, as depicted in Figure 2.

As a model of a single‐lane traffic, we assume as in Chen et al. [12] that both CAVs and HVs travel at a constant free‐flow speed of $u_{f}$ until they reach their respective critical spacings, below which they enter the car‐following mode. During the car‐following process, vehicles react in some specific fashion to a stimulus from the preceding vehicle without overtaking. In general, different reaction times are expected for different vehicle types. We will differentiate four different reaction times by letting $\Delta t_{1}$ denote the reaction time of a CAV following a CAV within a CAV platoon, $\Delta t_{2}$ to denote the reaction time of a CAV platoon leader following an HV, $\Delta t_{3}$ to denote the reaction time of an HV following a CAV, and $\Delta t_{4}$ to denote the reaction time of an HV following an HV. On assuming the traffic stream depicted in Figure 2 as a model for the whole traffic flow dynamics, we define the density *k* (veh/m) of the traffic stream as the average number of vehicles per unit length. Then, the critical density of the mixed traffic stream $k_{cri}$, at which all the vehicles travel in the car‐following mode at free‐flow speed $u_{f}$, is calculated as follows:

(1) $$\begin{array}{ccc} k_{cri} & = & \frac{m+n}{u_{f}\cdot \Delta t_{4}\cdot \left(m-1\right)+u_{f}\cdot \Delta t_{3}+u_{f}\cdot \Delta t_{1}\cdot \left(n-1\right)+u_{f}\cdot \Delta t_{2}+\left(m+n\right)\cdot d_{j}}\\ & = & \frac{m+n}{u_{f}\cdot \Delta t_{4}\cdot m+u_{f}\cdot \Delta t_{1}\cdot n+\left(m+n\right)\cdot d_{j}-u_{f}\cdot \Delta t_{4}+u_{f}\cdot \Delta t_{3}-u_{f}\cdot \Delta t_{1}+u_{f}\cdot \Delta t_{2}}\\ & = & \frac{1}{u_{f}\cdot \Delta t_{4}\cdot \frac{m}{m+n}+u_{f}\cdot \Delta t_{1}\cdot \frac{n}{m+n}+d_{j}+\frac{u_{f}}{m+n}\cdot \left(\Delta t_{2}+\Delta t_{3}-\Delta t_{1}-\Delta t_{4}\right)}\\ & = & \frac{1}{u_{f}\cdot \left[\Delta t_{4}\cdot \frac{m}{m+n}+\Delta t_{1}\cdot \frac{n}{m+n}+\frac{1}{m+n}\cdot \left(\Delta t_{2}+\Delta t_{3}-\Delta t_{1}-\Delta t_{4}\right)\right]+d_{j}}\\ & = & \frac{1}{u_{f}\cdot \left[\Delta t_{4}\cdot \left(1-p\right)+\Delta t_{1}\cdot p+\frac{p}{n}\cdot \left(\Delta t_{2}+\Delta t_{3}-\Delta t_{1}-\Delta t_{4}\right)\right]+d_{j}} \end{array}$$

where $p\;=\frac{n}{m+n}\;$denotes the CAV penetration rate in the mixed traffic stream and $d_{j}$ is the jam distance (including the vehicle length). We have assumed that each vehicle has the same jam distance since all the vehicles in this paper are cars with the same length. It can be inferred from Equation (1) that the critical density of a mixed traffic stream depends on the CAV penetration rate *p*, reaction time of different vehicle following types ($\Delta t_{1},\Delta t_{2},\Delta t_{3},\Delta t_{4}$), and CAV platoon size *n* (commonly referred to as the CAV platoon intensity).

**FIGURE 3:**
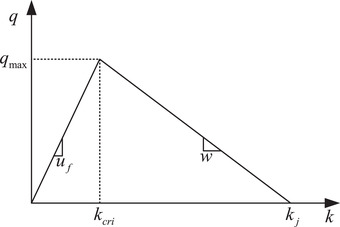
Triangular FD

The FD of traffic flow is a diagram that gives a relation between flow and density in equilibrium traffic, which is usually described by triangular shape. As illustrated in Figure 3, the triangular FD consists of two vectors, one positive and the other negative [41]. The flow‐density (q−k) relationship in the triangular FD can be expressed as Equation (2):

(2) $$q\;=\left\{\begin{array}{cc} u_{f}\cdot k & ifk\leq k_{cri}\\ w\cdot \left(k_{j}-k\right) & otherwise \end{array}\right.$$

where $u_{f}$ denotes the positive free‐flow speed, *w* denotes the negative backward wave speed, $k_{j}$ denotes the jam density, $k_{cri}$ represents the critical density that maximizes traffic flows, and the corresponding maximum flow is depicted as $q_{max}$ in Figure 3. Relating Equation (1) to Equation (2), the maximum flow of the mixed traffic stream $q_{max}$ is then expressed as follows:

(3) $$q_{max}=\frac{u_{f}}{u_{f}\cdot \left[\Delta t_{4}\cdot \left(1-p\right)+\Delta t_{1}\cdot p+\frac{p}{n}\cdot \left(\Delta t_{2}+\Delta t_{3}-\Delta t_{1}-\Delta t_{4}\right)\right]+d_{j}}\;$$

The backward wave speed of the mixed traffic stream *w* is then formulated as:

(4) $$\begin{array}{ccc} w & = & -\frac{q_{\max}}{k_{cri}-k_{j}}\\ & = & -\frac{\frac{u_{f}}{u_{f}\cdot \left[\Delta t_{4}\cdot \left(1-p\right)+\Delta t_{1}\cdot p+\frac{p}{n}\cdot \left(\Delta t_{2}+\Delta t_{3}-\Delta t_{1}-\Delta t_{4}\right)\right]+d_{j}}}{\frac{1}{u_{f}\cdot \left[\Delta t_{4}\cdot \left(1-p\right)+\Delta t_{1}\cdot p+\frac{p}{n}\cdot \left(\Delta t_{2}+\Delta t_{3}-\Delta t_{1}-\Delta t_{4}\right)\right]+d_{j}}-\frac{1}{d_{j}}}\\ & = & -\frac{d_{j}}{\Delta t_{1}\cdot p+\Delta t_{4}\cdot \left(1-p\right)+\frac{p}{n}\cdot \left(\Delta t_{2}+\Delta t_{3}-\Delta t_{1}-\Delta t_{4}\right)} \end{array}$$

It is clearly deduced that all the critical density $k_{cri}$, backward wave speed *w*, and maximum flow $q_{max}$ of the mixed traffic stream depend on the CAV penetration rate *p*, the reaction times of different vehicle following patterns ($\Delta t_{1},\Delta t_{2},\Delta t_{3},\Delta t_{4}$), and the CAV platoon intensity *n*.

### 3.2 Monte Carlo method for CAV platoon intensity

In the existing research, CAV platoon intensity and CAV penetration rate were usually regarded as two unrelated and independent variables that can affect the mixed traffic capacity [11, 12]. However, unless the CAV controlling algorithm predetermines a specific number of platoon size, the CAV platoon intensity should correlate with the CAV penetration rate.

**FIGURE 4:**
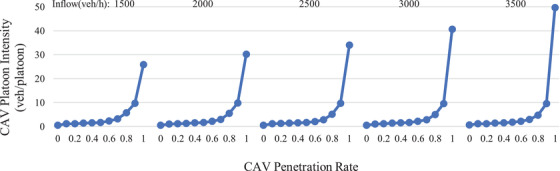
Simulated platoon intensity under different inflows and different CAV penetration rates

As in a previous simulation study by the authors [42] under different inflows (1500–3500 veh/h), CAV platoon intensities vary with CAV penetration rates (0.0–1.0), as illustrated in Figure 4. It can be observed that the CAV platoon intensity is considerably dependent on the CAV penetration rate.

In [42], the communication structure among CAVs was not studied; therefore, the CAV penetration of 1.0 generated a huge CAV platoon intensity, which was inconsistent with the limits of vehicular wireless communication range [43]. In order to meet the actual CAV communication range, this paper presets the maximum platoon intensity at a specific number. As suggested by [44], the maximum CAV platoon intensity is set at 20, below which the CAV platoon intensity depends on the CAV penetration rate. Furthermore, another interesting phenomenon can be explored in Figure 4, and, except for CAV penetration of 1.0, the increasing trends of CAV platoon intensity with the CAV penetration rate are similar for different inflows. Thus, in this study, inflow is not considered as an influential factor of CAV platoon intensity, but only the CAV penetration rate is regarded as a factor affecting the CAV platoon intensity.

**FIGURE 5:**
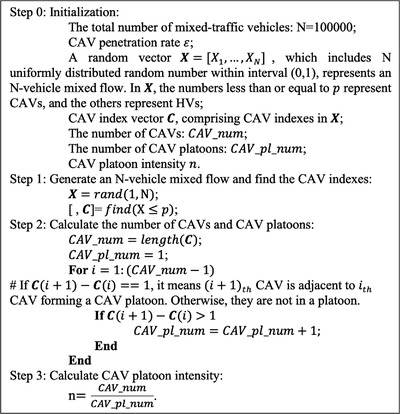
Monte Carlo algorithm

We have therefore employed the Monte Carlo Method to further study the relationship between the CAV platoon intensity *n* and CAV penetration rate *p*. For the Monte Carlo simulation, we have considered an infinite number of vehicles, including CAVs with penetration rate of *p* and HVs with penetration rate of 1−p, randomly distributed on a single‐lane link without overtaking or lane changing. In the case of traffic flow exceeding 1500 veh/h, adjacent CAVs are assumed to automatically form a platoon until the maximum size of a CAV platoon is achieved. The pseudocode for the Monte Carlo algorithm is elaborated in Figure 5.

**FIGURE 6:**
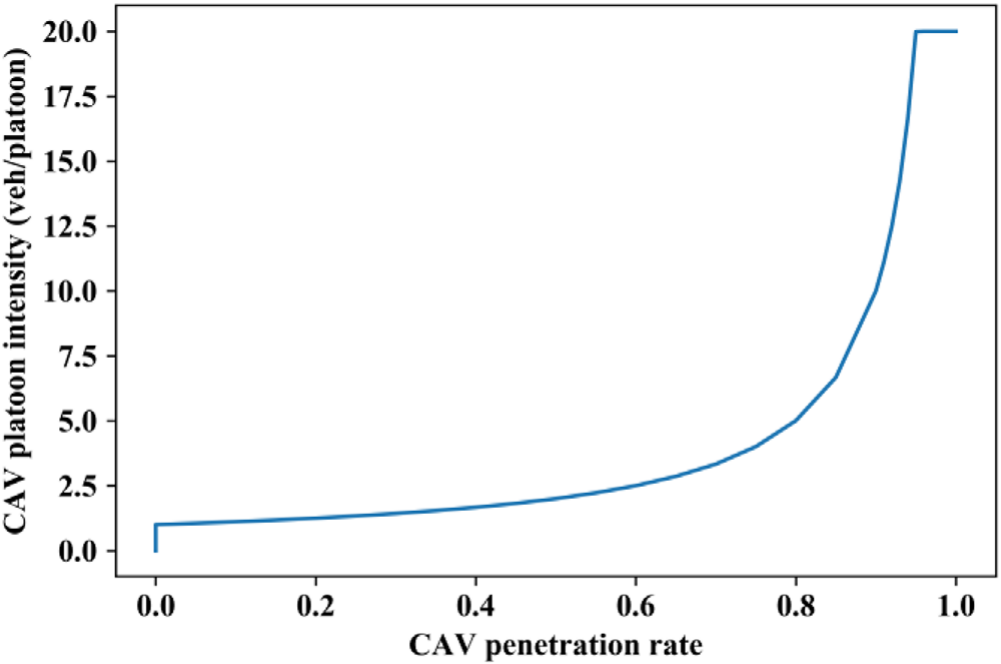
Fitting curve of Monte Carlo simulation result

The Monte Carlo simulation result is shown in Figure 6, where the relationship between CAV platoon intensity and CAV penetration rate perfectly fits the two‐term exponential model given by Equation (5). The sum of squared residuals (SSE) of the exponential fitting model is 1.0497, indicating a small random error of the fitting model. R‐square value is 0.9987, meaning that the exponential fit explains 99.87% of the total variation in the data about the average.

(5) $$n\;=\left\{\begin{array}{cc} 0 & p=0\\ 0.79\cdot e^{2.06p}+2.23\;\times \;10^{-8}\cdot e^{21.32p} & 0<p<0.96\\ 20 & 0.96\leq p\leq 1 \end{array}\right.$$

On substituting the CAV platoon intensity given by Equation (5) into Equations (3) and (4), the maximum flow and backward wave speed of the mixed traffic stream are reformulated as Equations (6) and (7).

(6) $$q_{max}=\left\{\begin{array}{c} \frac{u_{f}}{u_{f}\cdot \left[\Delta t_{4}\cdot \left(1-p\right)+\Delta t_{1}\cdot p+\frac{p\cdot \left(\Delta t_{2}+\Delta t_{3}-\Delta t_{1}-\Delta t_{4}\right)}{0.79\cdot e^{2.06p}+2.23\;\times \;10^{-8}\cdot e^{21.32p}}\right]+d_{j}}\;0\leq p<0.96\\ \frac{u_{f}}{u_{f}\cdot \left[\Delta t_{4}\cdot \left(1-p\right)+\Delta t_{1}\cdot p+\frac{p\cdot \left(\Delta t_{2}+\Delta t_{3}-\Delta t_{1}-\Delta t_{4}\right)}{20}\right]+d_{j}}\;0.96\leq p\leq 1 \end{array}\right.$$

(7) $$w\;=\left\{\begin{array}{c} -\frac{d_{j}}{\Delta t_{1}\cdot p+\Delta t_{4}\cdot \left(1-p\right)+\frac{p\cdot \left(\Delta t_{2}+\Delta t_{3}-\Delta t_{1}-\Delta t_{4}\right)}{0.7917\cdot e^{2.063p}+2.234\;\times \;10^{-8}\cdot e^{21.32p}}}\;0\leq p<0.96\\ -\frac{d_{j}}{\Delta t_{1}\cdot p+\Delta t_{4}\cdot \left(1-p\right)+\frac{p}{20}\cdot \left(\Delta t_{2}+\Delta t_{3}-\Delta t_{1}-\Delta t_{4}\right)}\;0.96\leq p\leq 1 \end{array}\right.$$

We notice that when 0≤p<0.96, the formula $\frac{p}{0.79\;\cdot \;e^{2.06p}\;+\;2.23\;\times \;10^{-8}\;\cdot \;e^{21.32p}}$ of Equation (6) is a non‐monotone non‐negative function that increases initially and then decreases with *p*. Therefore, the link maximum flow ($q_{max}$) of the mixed FD is not always an increasing function of CAV penetration rate but depends on the reaction times of different vehicle following patterns, especially relies on the sign of ($\Delta t_{2}+\Delta t_{3}-\Delta t_{1}-\Delta t_{4}$). When $\Delta t_{2}+\Delta t_{3}>\Delta t_{1}+\Delta t_{4}$, $q_{max}$ has the possibility to decrease at the initial stage and then increase with the CAV penetration rate. The impact of such vehicle reaction time setting ($\Delta t_{2}+\Delta t_{3}>\Delta t_{1}+\Delta t_{4}$) on link FD is counterintuitive and should be taken seriously.

### 3.3 MFD of a mixed homogeneous urban corridor

**FIGURE 7:**
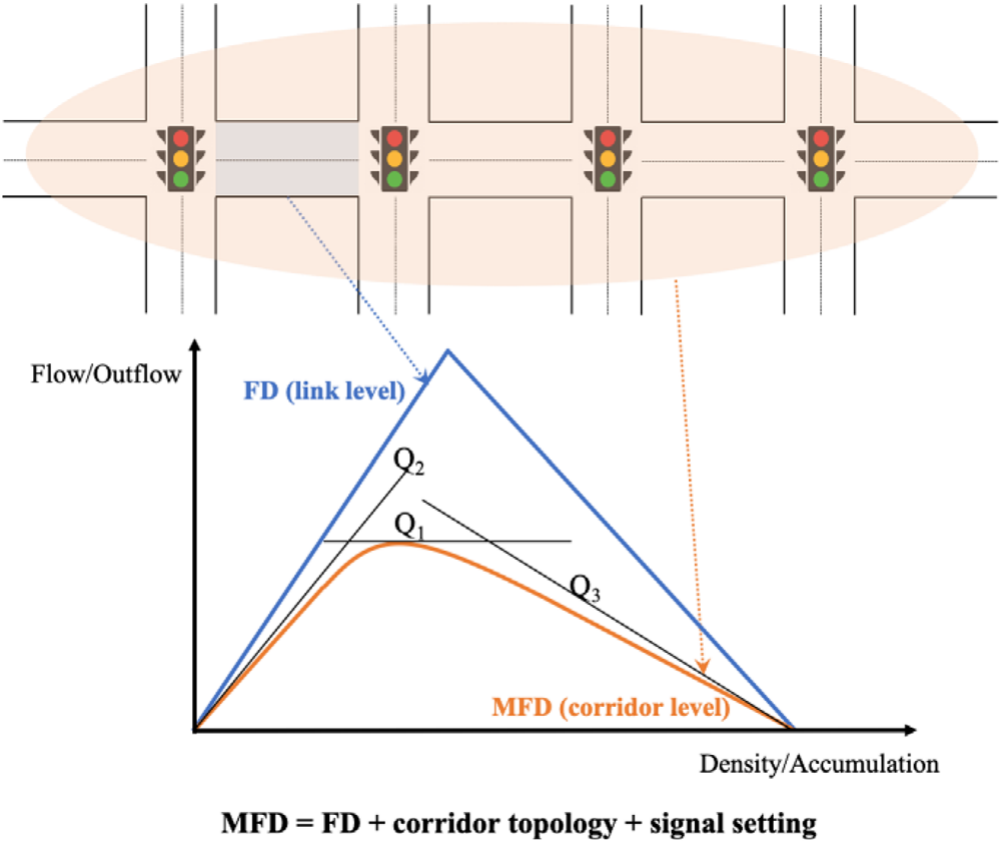
Principle of method of cuts

To estimate the MFD of the mixed corridor, method of cuts is adopted in this study. The principle of method of cuts is illustrated in Figure 7, and a corridor MFD can be obtained by the link FD, corridor topology, and signal setting. Given a mixed homogeneous corridor consisting of a series of successive links of uniform length *l*, each delimited by the same traffic signal settings of green time *G*, cycle time *C*, and offset δ, the corridor MFD is then only related to link FD. Thus, the mixed FD, including $q_{max}$ and *w* derived from the previous section, can be used to form the MFD of a mixed urban corridor.

As seen in Figure 7, the method of cuts aims to generate three families of upper envelopes ($Q_{1},Q_{2},Q_{3}$) that jointly bound the MFD from above. The three upper cuts for the mixed corridor MFD are formulated as follows:

(8) $$Q_{1}=q_{max}\;.\frac{G}{C}$$

where *Q*_1_ is the first family of cuts that uses a stationary observer at each signal intersection, $q_{max}$ is the maximum flow in the mixed FD, and *G* and *C* are the green and cycle time at each intersection, respectively.

(9) $$d_{\mathrm{forward}}=C\cdot \left(\mathrm{ceil}\left(\frac{\gamma_{\max}\left(\frac{l}{u_{f}}-\delta \right)}{C}\right)-\frac{\gamma_{\max}\left(\frac{l}{u_{f}}-\delta \right)}{C}\right)$$

(10) $$u_{forward}=\frac{\gamma_{max}\cdot l}{d_{forward}+\gamma_{max}\cdot \frac{l}{u_{f}}}\;$$

(11) $$f_{forward}=\frac{d_{forward}-C+G}{d_{forward}+\gamma_{max}\cdot \frac{l}{u_{f}}}\;$$

(12) $$Q_{2}=\;k\cdot u_{forward}+q_{max}\cdot f_{forward}$$

*Q*_2_ is the second family of cuts that takes into consideration an observer moving forward along the corridor at speed $u_{f}$. $\gamma_{max}$ denotes the maximum number of consecutive links that a forward observer can traverse before being stopped by a red signal light. *l* is the link length between consecutive intersections, δ is the offset of each traffic signal, $d_{forward}$ represents the delay at each stop of a forward‐moving observer, $u_{forward}$ is the average speed of the forward‐moving observer, and $f_{forward}$ is the fraction of time that the forward‐moving observer spends, artificially stopped, in green phases due to extended red phases.

(13) $$\delta_{w}=\;C-\delta$$

(14) $$d_{\mathrm{backward}}=C\cdot \left(\mathrm{ceil}\left(\frac{\gamma_{\max}\cdot \left(\frac{l}{w}-\delta_{w}\right)}{C}\right)-\frac{\gamma_{\max}\cdot \left(\frac{l}{w}-\delta_{w}\right)}{C}\right)$$

(15) $$u_{backward}=\frac{\gamma_{max}\cdot l}{d_{forward}+\gamma_{max}\cdot \frac{l}{w}}\;$$

(16) $$f_{backward}=\frac{d_{forward}-C+G}{d_{forward}+\gamma_{max}\cdot \frac{l}{w}}\;$$

(17) $$r\;=\frac{\left(u_{f}+w\right)\cdot q_{max}}{u_{f}}\;$$

(18) $$Q_{3}=\;-k\cdot u_{backward}+q_{max}\cdot f_{backward}+r\cdot \frac{u_{backward}}{w}$$

*Q*_3_ is the third family of cuts for an observer travelling backward along the corridor at speed *w*, $\delta_{w}$ is the signal offset from the backward direction, *r* is the maximum rate of the backward‐moving observer being passed, and $d_{backward}$, $u_{backward}$, and $f_{backward}$ are the delay, average speed, and fraction of time spent in extended red phases by the backward‐moving observer.

## 4 NUMERICAL EXPERIMENTS

### 4.1 Scenario setting

In this section, a numerical experiment is conducted in an urban corridor of San Francisco with similar scenario settings as referenced in [38]. The parameters used in estimating MFDs include the average link length l=122.9m, free‐flow speed $u_{f}=\;13.4\;m/s$, jam distance $d_{j}=\;7.7\;m$, green time of each signal G=21s, cycle time of each signal C=60s, offset of each signal δ=3s, and $\gamma_{max}=\;2$. Turning movements are not considered in our experiments.

As mentioned in Section 3.2, link's maximum flow $q_{max}$ is related to the sign of ($\Delta t_{2}+\Delta t_{3}-\Delta t_{1}-\Delta t_{4}$). Thus, when analyzing the corridor capacity, we also consider the sign of ($\Delta t_{2}+\Delta t_{3}-\Delta t_{1}-\Delta t_{4}$). The case study is divided into two scenarios: $\Delta t_{2}+\Delta t_{3}-\Delta t_{1}-\Delta t_{4}\leq 0\;$and $\Delta t_{2}+\Delta t_{3}-\Delta t_{1}-\Delta t_{4}>0$.

### 4.2 Analysis of corridor capacity

**FIGURE 8:**
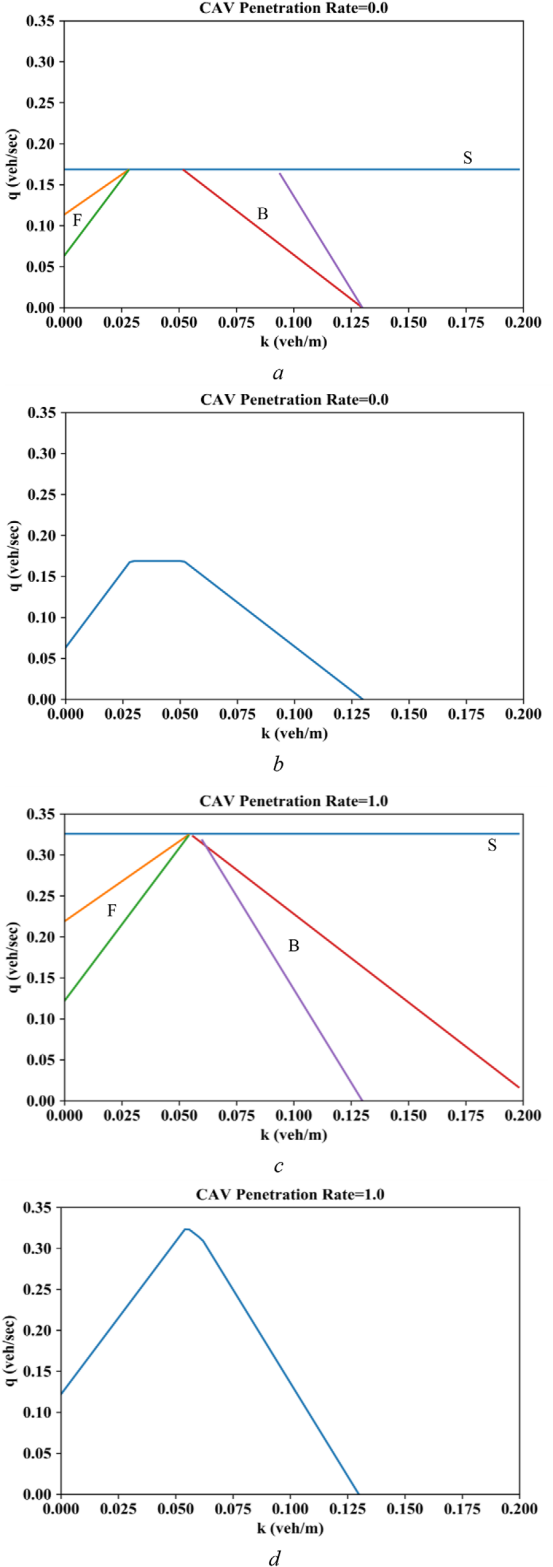
Analytical MFDs derived from three families of cuts. (a) Cuts for CAV penetration of 0.0. (b)Upper bound of the MFD when CAV penetration is 0.0. (c) Cuts for CAV penetration of 1.0. (d) Upper bound of the MFD when CAV penetration is 1.0

We first set the reaction time of a CAV following a CAV at 0.5 s ($\Delta \;t_{1}=\;0.5\;s$), a CAV following an HV at 0.9 s ($\Delta \;t_{2}=\;0.9\;s$), an HV following a CAV at 1.0 s ($\Delta \;t_{3}=\;1.0\;s$), and an HV following an HV at 1.5 s ($\Delta \;t_{4}=\;1.5\;s$). Thus, $\Delta t_{2}+\Delta t_{3}\leq \Delta t_{1}+\Delta t_{4}$ is satisfied. Given CAV penetration rate of 0.0, the maximum link flow and backward wave speed of the FD can be calculated and then be fed into cuts in Figure 8(a), where F means the cut is obtained by a forward‐moving observer, S represents a stationary observer, and B denotes a cut derived by a backward‐moving observer. As seen in Figure 8(b), the intersection of all cuts outlines the upper envelope of the MFD. Similarly, through the proposed analytical method, the cuts and upper envelope of the urban corridor MFD for CAV penetration rate of 1.0 are obtained, as shown in Figure 8(c) and (d). The peak of the MFD can be regarded as the capacity of the urban corridor. Therefore, by comparing Figure 8(b) and (d), we can conclude that replacing all the HVs with CAVs on the urban corridor almost doubles the capacity.

**FIGURE 9:**
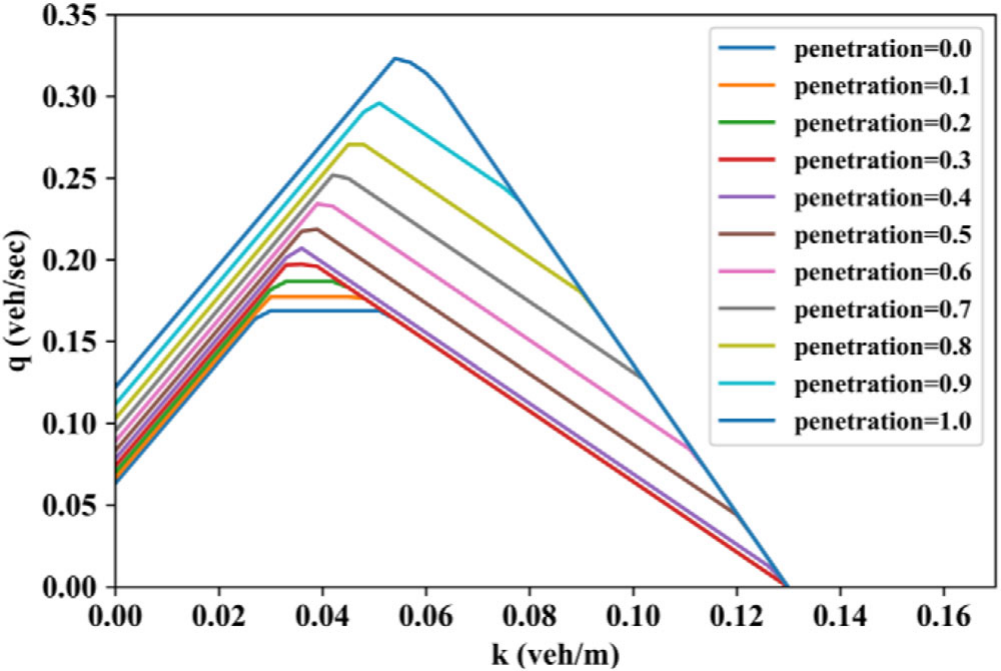
Analytical MFD evolution with CAV penetration when $\Delta t_{2}+\Delta t_{3}\leq \Delta t_{1}+\Delta t_{4}$

To comprehensively investigate the impact of CAV penetration rate on the mixed corridor MFD and capacity, the proposed analytical method is applied to a full spectra of CAV penetration rates ranging from 0.0 to 1.0 with an interval of 0.1, as depicted in Figure 9. We can decipher from Figure 9 that the corridor capacity increases with the CAV penetration rate, and the magnitude of capacity growth accelerates with the increase of CAV penetration.

### 4.3 Sensitivity to reaction time

**FIGURE 10:**
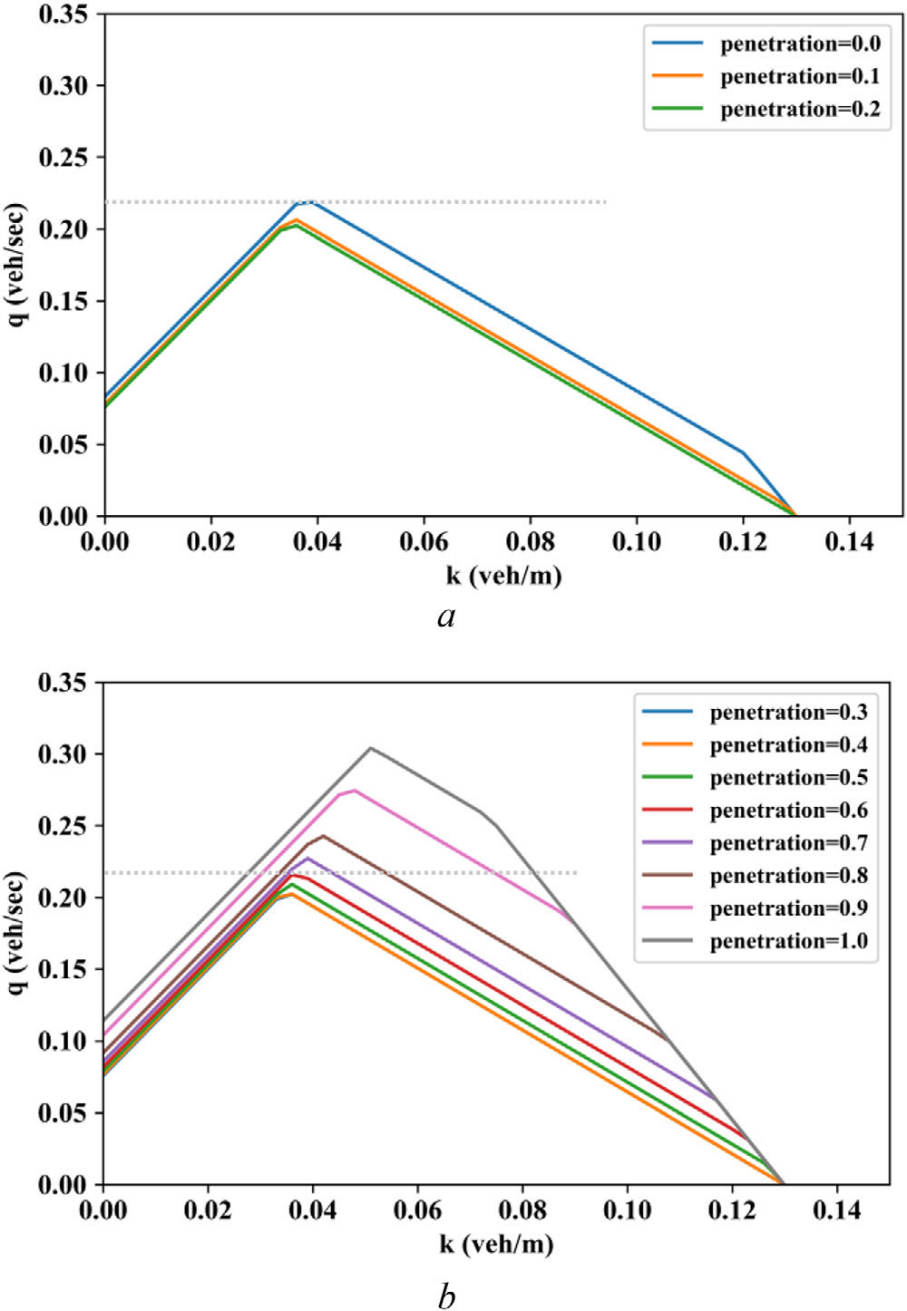
Analytical MFD evolution with CAV penetration rate when $\Delta t_{2}+\Delta t_{3}>\Delta t_{1}+\Delta t_{4}$. (a) CAV penetration rate from 0.0 to 0.2. (b) CAV penetration rate from 0.3 to 1.0

In this investigation, we set the reaction times of a CAV following a CAV at 0.5 s ($\Delta \;t_{1}=\;0.5\;s$), a CAV following an HV at 2.0 s ($\Delta \;t_{2}=\;2.0\;s$), an HV following a CAV at 1.0 s ($\Delta \;t_{3}=\;1.0\;s$), and an HV following an HV at 1.0 s ($\Delta \;t_{4}=\;1.0\;s$). Thus, $\Delta t_{2}+\Delta t_{3}>\Delta t_{1}+\Delta t_{4}$ is satisfied. Figure 10 shows the MFD evolution with CAV penetration rates ranging from 0.0 to 1.0. We note that the corridor capacity decreases when the CAV penetration rates increases from 0.0 to 0.2 [as seen in Figure 10(a)], then gradually recovers until the original level of corridor capacity is restored at CAV penetration rate of 0.6; thereafter, the corridor capacity increases with the growth of CAV penetration rate, as seen in Figure 10(b), where the CAV penetration rates of 0.2 and 0.6 can be treated as two critical turning points. In this case, the CAV technology is verified to be advantageous in corridor capacity only when CAV penetration rate is larger than 0.6, and low penetration of CAVs even results in a decline in corridor capacity. This analytical result has demonstrated that higher CAV penetration rates may not always lead to greater mixed traffic capacity of urban corridors. Such observation informs traffic planners and managers to be mindful of different CAV technologies in the mixed urban environment. If the reaction times of different vehicle following patterns are not properly handled, the introduction of CAVs may have a negative effect on corridor capacity.

We can also discerned that with the exception of the reaction times $\Delta t_{1}$, $\Delta t_{2}$, $\Delta t_{3}$, and $\Delta t_{4}$, the parameter settings of both the urban corridor and vehicle behaviors are the same in Figures 9 and 10. However, the MFDs of each CAV penetration rate in the two scenarios are distinct. This observation shows that both the evolution trend as well as the size of the corridor capacity can be affected by the reaction time settings of different vehicle following patterns.

### 4.4 Comparison with simulation

**FIGURE 11:**
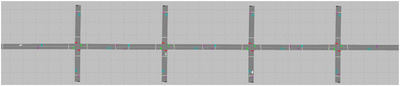
Corridor simulation in VISSIM

In this section, we will compare the proposed analytical approximation of the corridor MFD with the traffic simulation data obtained by PTV VISSIM [45]. As depicted in Figure 11, the urban corridor consists of four signal intersections and the simulation is conducted in VISSIM 2020 [46]. The scenario setting is the same as that in the analytical experiment in Section 4.1 (based on a San Francisco corridor). The length of each link l=122.9m, the free‐flow speed $u_{f}=\;13.4\;m/s$, the jam distance $d_{j}=\;7.7\;m$, the green time of each signal G=21s, the cycle time of each signal C=60s, and the offset of each signal δ=3s. In order to satisfy the jam distance of 7.7 m, in addition to the vehicle length of 4.5 m, we set the standstill distance of vehicles in VISSIM to 3.2 m. Furthermore, we have created two vehicle types—named “CAV” and “HV”—and simulated their driving behavior using the default car following model in VISSIM. The parameters are then customized according to vehicle reaction times. Under “CAV” driving behavior, the headway time of a CAV following a CAV (“CC1” in VISSIM) is set at 0.5 s, and the headway time of a CAV following an HV (“W99cc1Distr” in VISSIM) is set at 2.0 s. The longitudinal oscillation of a CAV following a CAV (“CC2” in VISSIM) is set at 0, indicating more stable following behaviors between consecutive CAVs. Furthermore, there is a new module called “Autonomous Driving” in VISSIM 2020, where we choose the platooning function for “CAV” and set the maximum platoon size as 20. Under “HV” driving behavior, the headway time of an HV following an HV (“CC1” in VISSIM) is set at 1.0 s, and the headway time of an HV following a CAV (“W99cc1Distr” in VISSIM) is set at 1.0 s. The other parameters not mentioned here remain at the default values in VISSIM. Furthermore, to test the influence of CAV penetration rate on the corridor capacity, a Python script is used to communicate with VISSIM through COM interface, traversing the CAV penetration rate from 0.0 to 1.0 at increments of 0.1.

**FIGURE 12:**
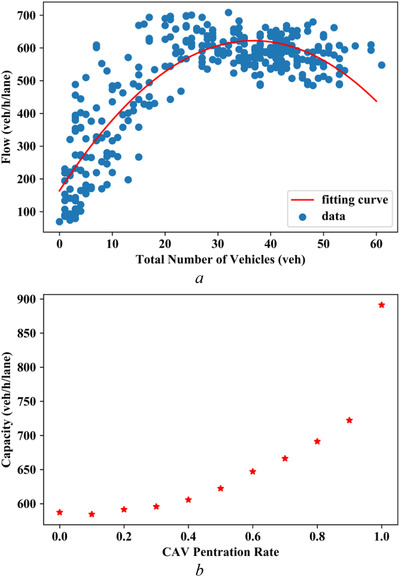
Simulated MFD evolution with CAV penetration when $\Delta t_{2}+\Delta t_{3}>\Delta t_{1}+\Delta t_{4}$. (a) Simulation data fitting to the MFD. (b) Fitted capacity for CAV penetration rate from 0.0 to 1.0

During each simulation, the total number of vehicles and traffic flow are aggregated, as shown in Figure 12(a), and then, fitted with a parabolic curve which is regarded as an MFD [47, 48]. The corridor capacity can be identified by the cusp in the MFD curve. With the setting of $\Delta t_{2}+\Delta t_{3}>\Delta t_{1}+\Delta t_{4}$ ($\Delta \;t_{1}=\;0.5\;s$, $\Delta \;t_{2}=\;2.0\;s$, $\Delta \;t_{3}=\;1.0\;s$, $\Delta \;t_{4}=\;1.0\;s$), the capacity evolution with CAV penetration rate is presented in Figure 12(b). The corridor capacity experiences a slight drop when CAV penetration rate increases from 0.0 to 0.1, then a slight increase of capacity is observed when CAV penetration rate increases from 0.2 to 0.3. For under 30% penetration rate of CAVs, the corridor capacities are almost at the even level. For over 30% penetration rate of CAVs, the high changes in corridor capacity becomes more prominent with increase in CAV penetration rates. The CAV penetration rates of 0.1 and 0.3 are critical turning points in the simulation. Although the specific turning points exhibit some differences between the analytical experiment and VISSIM simulation, the overall evolution trends of corridor capacity with CAV penetration rates are similar. The difference may be attributed to the randomness of VISSIM simulation, and, moreover, the analytical experiment only produces the upper envelope of MFD, while the VISSIM simulation produces MFD.

## 5 CONCLUSIONS

This paper is the first study to develop an analytical methodology to study how the macroscopic capacity of an urban corridor evolves with the penetration of CAVs in transportation systems. Initially, the link capacity and backward wave speed of a mixed traffic stream were formulated and described as a mixed FD, taking the reaction times of different vehicle following patterns, CAV penetration rates, and CAV platoon intensities into consideration. Particularly, through Monte Carlo simulations, the CAV platoon intensity was expressed as an exponential function of CAV penetration rate. Based on the mixed FD, the upper bound of an MFD for an urban corridor with mixed traffic was derived theoretically through the method of cuts. Then, two numerical experiments were carried out to implement the proposed methodology. The first experimental results revealed a monotonic increasing relationship between corridor capacity and CAV penetration rate for $\Delta t_{2}+\Delta t_{3}\leq \Delta t_{1}+\Delta t_{4}$. However, when $\Delta t_{2}+\Delta t_{3}>\Delta t_{1}+\Delta t_{4}$ in the second experiment, the corridor capacity suffered slight reduction for low CAV penetrations, indicating that higher CAV penetration rates may not always yield a greater mixed traffic capacity of urban corridors. In addition, different reaction time settings ($\Delta t_{1}$, $\Delta t_{2}$, $\Delta t_{3}$, $\Delta t_{4}$) yielded different MFDs for the same corridor. Finally, simulation tests were conducted in VISSIM 2020 to validate the usefulness of the proposed analytical method on mixed traffic corridors. The variation trend of corridor capacity with CAV penetration rate is similar between simulation results and analytical results. Slightly different is that the turning point of simulation results is 0.1, while the turning point of analytical results is 0.2.

The implication of the proposed theoretical framework can be beneficial for car manufacturers to determine the reaction time settings of CAVs. Also, it can help traffic authorities to pay attention to the urban traffic condition when the CAV penetration rate is low, and to develop traffic demand management strategies under mixed flow. The limitation of this paper lies in the assumption that the corridor traffic flow only includes CAV and HV and does not include pedestrians or cyclists. This study can be extended in several directions in the future. First, this study adopted method of cuts to draw the upper bounds of MFDs; how the other analytical methods for MFDs would perform in mixed traffic corridors is also of interest. Second, this paper is focused on analytical MFDs in urban corridors, which can be further extended to urban networks.
